# Human Mobility Restrictions and COVID-19 Infection Rates: Analysis of Mobility Data and Coronavirus Spread in Poland and Portugal

**DOI:** 10.3390/ijerph192114455

**Published:** 2022-11-04

**Authors:** Artur Strzelecki, Ana Azevedo, Mariia Rizun, Paulina Rutecka, Kacper Zagała, Karina Cicha, Alexandra Albuquerque

**Affiliations:** 1 Department of Informatics, University of Economics in Katowice, 40-287 Katowice, Poland; 2 CEOS.PP, Porto Accounting and Business School, Polytechnic Institute of Porto, 4200-465 Porto, Portugal; 3 Department of Communication Design and Analysis, University of Economics in Katowice, 40-287 Katowice, Poland

**Keywords:** COVID-19, human mobility, state restrictions, lockdown, population behavior, coronavirus infection rates

## Abstract

This study examines the possibility of correlation between the data on human mobility restrictions and the COVID-19 infection rates in two European countries: Poland and Portugal. The aim of this study is to verify the correlation and causation between mobility changes and the infection spread as well as to investigate the impact of the introduced restrictions on changes in human mobility. The data were obtained from Google Community Mobility Reports, Apple Mobility Trends Reports, and The Humanitarian Data Exchange along with other reports published online. All the data were organized in one dataset, and three groups of variables were distinguished: restrictions, mobility, and intensity of the disease. The causal-comparative research design method is used for this study. The results show that in both countries the state restrictions reduced human mobility, with the strongest impact in places related to retail and recreation, grocery, pharmacy, and transit stations. At the same time, the data show that the increase in restrictions had strong positive correlation with stays in residential places both in Poland and Portugal.

## 1. Introduction

We observe a different dynamic fluctuation in the number of confirmed COVID-19 cases in the year 2022, albeit scientists are continuously researching the SARS-CoV-2 virus, methods of its prevention, and treatment. Despite the vaccinations and medications being developed, it is still necessary to find effective ways to limit the transmission of the virus. Not everyone is vaccinated, and some people cannot be due to the health conditions and possible side effects of the contraindications. In addition, the number of cases remains significant despite the number of vaccinated people. Before vaccinations and medications had been developed, the only form of counteraction was establishing regulations and recommendations regarding population behavior and mobility reductions. Regulations and recommendations, such as limiting business activity in selected sectors, limiting gatherings, restrictions on mobility, or covering the nose and mouth, were introduced and lifted by the governments of countries depending on the current epidemic situation.

Each government is simultaneously struggling with its internal problems and the attitudes of its citizens and managing the restrictions in the best possible way for its interests while considering many factors, such as the number of cases of disease, healthcare efficiency, climate change, the culture of the country, the ability of the economy to survive, and many more.

Restricting movement appears to be logically the most appropriate way to counteract viral transmission. However, it implies a lot of inconvenience and difficulties. Restrictions on the number of people in stores, sports, cultural centers, restaurants, hotels, and others, had a significant impact on the bottom line. The need to support business with subsidies has exposed governments to considerable expenses. Despite this, it was not always possible to save them. In the face of uncertainty as to when the pandemic will end, research into compliance with mobility restrictions and their impact on the number of cases and on the economy is particularly important.

Unlimited communication possibilities offered by the Internet allow for the free exchange of views between people living in different countries. They represent different attitudes and cultures, have different levels of education and are susceptible to media messages to different extents. This communication capability allows Internet users (e.g., on social media) to comment on government regulations. They compare governmental regulations and recommendations and criticize these actions. There is also an increasing number of unsubstantiated theories that are spread online [1,2,3]. So, it is also important to provide solid evidence that can help governments convince citizens to comply with the restrictions and verify the effectiveness of this method in light of the epidemic situation over the past two years.

In different countries, citizens comply with the restrictions in different ways. It may be the result of many factors, such as the level of trust in the government [4], the level of democracy in the country [5], the health literacy of citizens [6], or the political views of citizens [7]. The quality of social campaigns is also of great importance. These campaigns aim at informing citizens about the restrictions that are being introduced and their reasons [4].

The lack of scientific evidence that supports the effectiveness of specific restrictions against the pandemic can lead to questioning regarding their reliability. Examples of such objections are very visible, for instance, in Poland. Due to the restrictions, many sectors of the economy were closed in this country during the pandemic. During the period of the first lockdown (March–April 2020), it was clear that most shareholders understood and endeavored to accept the restrictions. However, in autumn 2020, new regulations established to restrict economic activity led to numerous strikes. They were caused, among others, by the lack of communication and explanation of the reasons why the restriction was introduced. Entrepreneurs did not accept the unproven reasons why some economic sectors should close, while the others should not. The cases of social opposition, such as the above-mentioned strikes of entrepreneurs in Poland, significantly hindered the prevention of the pandemic.

The current research gap is represented by a small number of studies that prove beyond any doubt the correlation between changes in human mobility with the effectiveness of nonpharmaceutical interventions (especially mobility restrictions) in counteracting the spread of infectious disease epidemics. The motivation behind the present study is to fill the current research gap by exploring the influence of mobility restrictions as a nonpharmaceutical intervention, especially mobility restrictions, on the spread of COVID-19. An additional objective of the proposed study is to examine the effectiveness of restrictions on mobility introduced by the Governments of two individual European countries, namely Portugal and Poland, during the COVID-19 pandemic to identify the correlation between human mobility and infection rate. On the one hand, we selected these two countries as a convenient sample and on the other hand, because they have significantly different COVID-19 vaccination rates.

The objective of this study is to (a) identify the correlation between mobility changes and the infection spread as well as (b) investigate the impact of the introduced restrictions on changes in human mobility. The study will be supported by data from Google Mobility Reports, Apple Mobility Trends Reports, and The Humanitarian Data Exchange.

## 2. Research Background

The COVID-19 pandemic is an unprecedented phenomenon in modern world history. The introduction of low-cost airlines, the availability of comfortable cars and trains, and the growing internationalization of business facilitated the mobility of the world population far more than during previous pandemics. According to the findings of researchers, factors such as: high human mobility [8], the high rate of reproduction of the virus, and the droplet and airborne pathway of infection contribute to the rapid spread of this virus worldwide [9]. At the same time, the recognition of COVID-19 as a pandemic on 11 March 2020 had a significant impact on many areas of social activities. Due to the pandemic, the governments of particular countries decided to introduce restrictions, among others, on the functioning of the selected branches of the economy, on schools, or on movement. These activities are referred to as nonpharmaceutical interventions (NPI), and they aim at reducing the spread of the virus [10].

The introduced restrictions on movement, aimed at increasing “social distancing” and “physical distancing”, have been voluntarily undertaken or introduced by national governments in the form of a temporary law during the pandemic [11] and had a significant impact on trends in movement, both nationally and worldwide. Strong restrictions on mobility, such as those adopted at the beginning of the pandemic in China (especially in the Wuhan and Hubei provinces), led to a sharp stoppage in the number of symptomatic patients and calmed down the epidemic in that particular region. However, the Chinese government also considered that patients with no symptoms or with mild symptoms could have also been carriers of the virus and, therefore, they were banned from traveling [12].

The necessity for governments to take decisions to impose restrictions on civil liberties has created the need for information on the mobility of citizens among countries. On 8 April 2020, the European Commission asked the European mobile network operators for anonymous data on the location of mobile phones. These data allowed the understanding, among other matters, how the movement of citizens influences the development of the pandemic. The data can also be used to simulate and create epidemic models, verifying the impact of the introduced restrictions on the course of the pandemic [13]. Therefore, their complete anonymity was a condition for compliance with the EU privacy policy.

The restrictions introduced by the governments of different countries were introduced to different extents and at different times [14]. The measurement of the result of these differences is an additional difficulty related to the fact that citizens of some countries had the opportunity to move, including internationally, while others did not. According to the estimation from 3 May 2020, about one third of the world population has been restricted in movement [15]. While the viral transmission decreased after the restrictions had been introduced, the costs of such restrictions were enormous and had a negative impact on the global economy. Restrictions were also perceived differently by citizens in different countries who adopted government recommendations to varying degrees [14]. Many people were against the restrictions, understanding them as personal freedom limitations and the cause of economic deterioration. At the same time, others questioned the effectiveness of such activities [8] or considered them illogical. It is very difficult to measure how the restrictions contributed to the fight against the virus and, above all, which of them brought positive results and why. The reaction towards the restrictions was undoubtedly influenced by the way in which the messages were conveyed and the trust in the government that the citizens of a given country had, as well as the quality of the organized social campaigns influencing the perception of the pandemic [4]. It has also been shown that the political views presented in a given region have an impact on compliance with government restrictions [7].

As noted by the Chinese scientists who have studied the impact of reduced mobility on the spread of the SARS-CoV2 virus in China, it is difficult to separate the impact of human mobility from other potential factors, such as panic, and the virus effect, as well as the periodic intensification of movement and organization of gatherings related, for instance, to holidays [8]. Different countries reacted at different times during the pandemic. In Spain, Belgium, Italy, and the United Kingdom, governments delayed the lockdown for more than a month after the first case was discovered. In other countries, e.g., Poland, Portugal, Norway, and Denmark, lockdown was very quickly introduced: in Poland and Portugal, 9 days after the first case was confirmed [16] and 15 days after [5] in Norway and Denmark.

Studies have been undertaken to verify the correlation between the transmission of the virus and imposed restrictions, including NPI ones. Those studies used data on the numbers of those infected gathered in particular countries or regions [15]. However, it is indicated that the conclusions of these studies are mixed, and it is difficult to relate them to a global level because they only concerned a specific region, e.g., Wuhan, Florence [8,10], or a country, e.g., China, Switzerland, England, Australia, United States, Poland, Portugal, or Italy [17,18,19,20,21,22,23,24]. Few studies covered larger areas, for instance, bordering countries or a continent [5]. Additionally, in some studies, the selection of countries seems random, and most likely, it results from the data availability. In addition, Zhu, Mishra, and Virani tried to compare the data on the incidence of the disease with the information on the restrictions introduced by the governments of different countries at a certain time [14].

One of the more interesting studies is the comparison of mobility data with the estimates of daily transmission rates in 87 countries [15]. However, this study only covers the short, selected period from 21 February to 11 April 2020. Elsewhere, studies of the impact of mobility changes on the number of COVID-19 cases were undertaken [25]. It was also considered how quickly the country introduced the so-called lockdown and how it was related to the number of cases of disease and deaths [5]. Although the literature on mobility changes related to the COVID-19 pandemic is quite extensive so far, this topic can still benefit from the analysis of many different researchers. Maloney and Taskin analyzed whether changes in mobility result from restrictions imposed by governments or are voluntary [26]. An international team of researchers from the USA and Canada worked on an extensive analysis, which additionally took into account economic factors such as the decline in GDP and the democracy index [27]. The analysis of this team indicates that the greatest decrease in mobility occurred in South America, Western Europe, and New Zealand, particularly in areas such as retail, park attendance, and transit. In the early stages of the pandemic, China also studied changes in human mobility connected with the Chinese January holiday season (almost 40 days), which is associated with almost the largest annual human movement in the world [28]. These studies, however, did not show a significant impact of traveling within the holiday season on the worsening of the COVID-19 pandemic. Arora et al. studied the impact of the pandemic on changes in the natural environment, the reduction in air and water pollution, and nature restoration [29]. Part of the research focuses on the social aspect and the translation of the demographic characteristics of residents into reduced mobility. It was indicated that areas inhabited by a greater number of people over 65 years of age have a higher rate of reduced mobility than others [30].

Wang, Wei, Lin, and Li investigated the correlation between people’s awareness of the pandemic and mobility patterns [23], noting that people limit their mobility two weeks after learning about the situation. Lee et al. found that in higher-income communities more people chose to stay at home [31].

Based on the above-stated, the research questions (RQs) related to mobility changes during the COVID-19 pandemic are as follows:

RQ1: How and to what extent did restrictions in migration affect human mobility?

RQ2: Did the imposed restrictions affect the intensity of new cases of COVID-19 infection and to what extent?

RQ3: How and to what extent has the use of public places (such as commercial and recreational facilities, grocery stores and pharmacies, parks, and stations and stops) and workplaces changed because of the restrictions?

RQ4: How has the use of private places, such as places of residence, changed?

The research will be presented following three steps. Starting with the data collection, we will further proceed by structuring and analyzing the data with the objective of creating a single large dataset of available data on human mobility in the time of the COVID-19 pandemic. Finally, the generated dataset will be applied to the modeling of the new case statistics in correlation with the data on human mobility.

## 3. Materials and Methods

We used a causal-comparative research design [32] for this study. It is a research design that seeks to find relationships between independent and dependent variables after an action or event has already occurred. The goal is to determine whether the independent variable affected the outcome or the dependent variable, by comparing two or more groups of individuals. Causal-comparative research is also referred to as ex post facto research. Causal-comparative research designs attempt to determine relationships among variables but do not allow for the actual manipulation of these variables.

**Figure 1:**
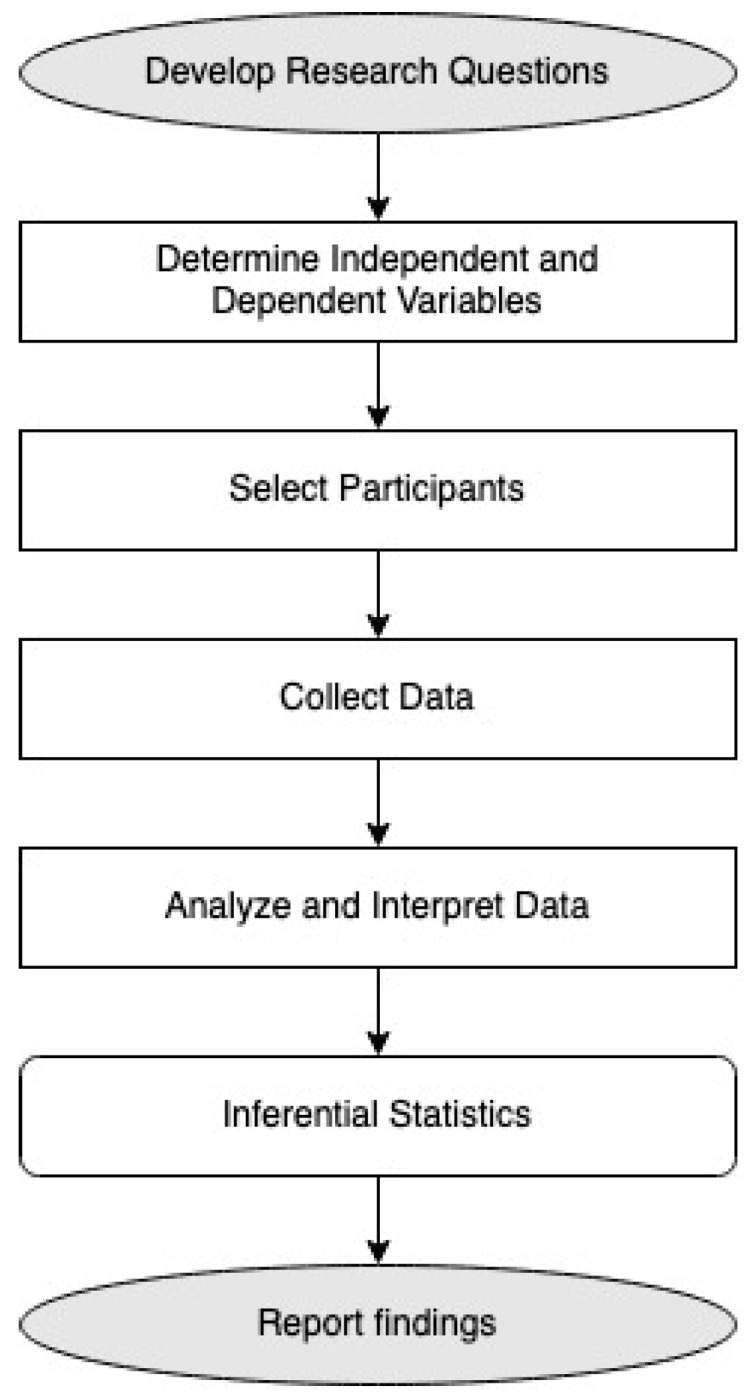
Process of casual-comparative design research.

Causal-comparative research typically compares two or more groups of subjects. Research subjects are generally split into groups based on the independent variable that is the focus of the study. The goal of the research is to determine what effect the independent variable may or may not have on the dependent variable or variables. In causal-comparative research, we investigate the impact of an independent variable on a dependent variable by comparing two groups of individuals. In our case, these are data coming from two different countries. Causal-comparative research occurs after the event or action has been completed. We are studying publicly available data. It is a retrospective way of determining what may have caused something to occur. In causal-comparative research, the subjects are already in groups because the action or event has already happened. Once the data have been collected, we analyze and interpret the results using inferential statistics. We are using Spearman’s Rho correlation and Granger causality. Figure 1 presents our research process.

The countries were selected as a convenient sample that could be the subject of the study: the home countries of the authors. This parameter was, therefore, incredibly prompt, uncomplicated, and economical. We decided to investigate just two countries, Poland and Portugal because the situation is multifaceted and very complex. The choice of countries was therefore associated with the possibility of a proper understanding of the data in context; therefore, the countries of the authors were examined. Not all information on what actually happened in countries during the pandemic and closure is available. It is not always possible to know what the decisions of governments were and what media messages were published. In addition, not everything can be inferred from the data, and not all the data we would like to obtain are available. Therefore, in order to draw the right conclusions, we analyzed them in the context of the countries where the authors live as observers of the phenomenon on the spot. Moreover, it allowed for determining whether the method would be appropriate for undertaking larger studies for a larger number of countries.

In a previous work comparing Poland and Portugal, we studied the correlation between the spread of COVID-19 and the interest in personal protective measures at the beginning of the pandemic [16]. In that work the interest in information about personal protective equipment was investigated using the following search terms in Polish and Portuguese: masks, antibacterial, and disinfection. This comparison in two languages was fruitful because it showed that the same interest could be expressed through different search terms, which were linked to cultural and social conditions. There was also a method adapted from the literature to study the correlation in Google Trends using day lags and machine learning to find clusters, which are groups of similar days in which interest in personal protective equipment belonged to the same clusters [16]. In the present study, we would now like to show the correlation between the data on mobility and the data on introduced restrictions and the correlation between restrictions, mobility changes, and the number of new disease cases in these two countries.

### 3.1. Data Sources

We have accessed several statistical datasets and reports (to be enumerated further) presenting the situation on COVID-19 spread and its influence on the lives of people, in particular on their mobility. As the world is still struggling with the same problem, most of the research (surveys, statistical analyses, among others) conducted on the topic of the coronavirus are made freely available in PubMed Central, WHO COVID database, and many other publicly funded repositories, with permission to reuse with acknowledgment of the original source [33].

The data available from Google (Google LLC., Mountain View, CA, USA) report “COVID-19 and Changes in Migration Trends” show migration trends between 15 February 2020 until now. The data are structured by geographic regions and different place categories: shopping and leisure facilities, grocery and pharmacy stores, parks, stations and stops, places of work, and places of residence. The data contained in the report are prepared based on summarized, anonymized data sets from users who have activated the location history setting on a mobile device with the Android operating system. Yet, identification of users is not possible at any stage; the view of locations, contacts, or routes of individual users is also unavailable. The report contains data from 135 countries and is accessible to anyone. The data are of particular interest and use for the institutions responsible for health policy in their struggle with the COVID-19 pandemic. The report from Google, updated daily, is available in CSV format at [34].

Apple (Apple Inc., Cupertino, CA, USA) has shared the “COVID-19 Mobility Trends Report” showing the relative number of requests for driving, walking, or transit directions in a given country/region, subregion, or city in the Apple Maps service compared to a baseline on 13 January 2020. The data in the “COVID-19 Mobility Trends Report” were updated and were available until the 14 April 2022. The dataset is available in CSV format at [35]. The data provided by Apple contain the relative number of requests for moving directions.

The Humanitarian Data Exchange portal [36] presents a few datasets that can contribute to the research: the COVID-19 Global Travel Restrictions and Airline Information and the Global School Closures COVID-19. The datasets are available for download in the form of CSV and XLSX files. COVID-19 Government Measures Dataset, presented by the Humanitarian Data Exchange portal, belongs to the same category of datasets. It presents information in five categories: social distancing, movement restrictions, public health measures, social and economic measures, lockdowns. The data are presented in the form of a world map with colors changing depending on the selected criteria. The dataset can also be downloaded (XLSX file) [36].

Our final dataset was created based on the sources as mentioned earlier. In detail, we have used the following data:

1. Intensity of the disease

The file source is https://data.humdata.org/dataset/total-covid-19-tests-performed-by-country, accessed on 15 April 2022, and the file name is owid-covid-data.xlsx. This file contains total cases, deaths, and testing dataset alongside detailed source descriptions. The data of Poland and Portugal were extracted.

2. Vaccination

The source of the file is https://data.humdata.org/dataset/covid-19-vaccinations, accessed on 15 April 2022, and the file name is vaccination-data.csv. The file contains data about vaccination from all of the countries. Poland and Portugal were extracted.

3. Google Mobility Data

The source of the file is https://www.google.com/covid19/mobility/, accessed on 15 April 2022, and the file name is Region_Mobility_Report_CSVs.zip. The file contains data about mobility in six types of places. The final dataset has filtered data only about Poland and Portugal and is divided into years.

4. Apple Mobility Report

The source of the file is https://github.com/ActiveConclusion/COVID19_mobility/tree/master/apple_reports, accessed on 15 April 2022, as Apple stopped providing the data after 15 April 2022. We have used data from applemobilitytrends.csv and extracted data for Poland and Portugal.

Table 1 presents the process of extraction indicators from the sources and assigns them into four groups: vaccination, restrictions, mobility, and intensity.

### 3.2. Data Analysis

MsExcel^TM^ was used to organize the data taken from the several data sources referred to above in a dataset that comprised three files. One of the files contained all the data for the two countries. The other two files contained all the data for each country separately, one file for Poland and one file for Portugal. These were used to obtain the correlations necessary to answer the research questions. Data analysis was performed using the software Microsoft SPSS^TM^ (version 26). Data were imported to three SPSS^TM^ files from MsExcel^TM^.

To ascertain the differences in vaccination rates between the 2 countries the available variables that were normalized per hundred or per million were considered, namely, total_vaccinations_per_hundred, people_vaccinated_per_hundred, people_fully_vaccinated_per_hundred, total_boosters_per_hundred, and new_vaccinations_smoothed_per_million. The means of these variables were compared using the t-test for equality of means for independent samples.

Concerning the correlations, three groups of variables were identified/distinguished: (i) the ones indicating restrictions, (ii) the ones indicating mobility, and (iii) the ones indicating intensity of the disease.

1. The variables considered as indicating restrictions were stringency_index and new_tests_smoothed.
2. The variables considered as indicating mobility were driving, walking, retail_and_recreation, grocery_and_pharmacy, parks, transit_stations, workplaces, and residential.
3. The variables considered as indicating intensity of the disease were new cases, new deaths, reproduction rate, and hosp_patients.

Four types of correlation were considered for each country: (i) between the variables belonging to the same group, (ii) between variables indicating restrictions and mobility, (iii) between variables indicating restrictions and intensity of the disease, and (iv) between variables indicating mobility and intensity of the disease.

Correlations were obtained using the Spearman’s rank correlation coefficient ($r_{s}$) because it allows the identification of monotonic relations between two variables, not just linear relations [37]. Causations were calculated with the use of Granger causality test, calculating F-statistics and *p*-values [38]. This test was calculated with the RealStatistics Resource Pack for MsExcel^TM^.

## 4. Results

Concerning the differences between the vaccination rates in Poland and in Portugal, we can verify that the means of the considered vaccination variables are higher for Portugal, pointing to higher vaccination rates in Portugal (Table 2). The *t*-test for the equality of means of the independent samples reveals that the differences between the two countries are very significant for the variables total_vaccinations_per_hundred (*t* = −4.056; sig = 0.000), people_fully_vaccinated_per_hundred (*t* = −0.487; sig = 0.000), and new_vaccinations_smoothed_per_million (*t* = −11.466; sig = 0.000) and not very significant for the variable total_boosters_per_hundred (*t* = −2.095; sig = 0.037). Thus, we can establish that vaccination rates were different among the two countries.

### 4.1. Correlations between Variables Belonging to the Same Type

Considering the variables belonging to the group restrictions in Poland, there is no correlation between the two variables stringency_index and new_tests_smoothed ($r_{s}=0.060$; sig = 0.00). Considering the variables belonging to the group mobility in Poland (see Appendix A, Table A1), all the correlations are highly significant. We observe that there are strong to moderate correlations between all the variables, with the exception of a weak correlation between the variables workplaces and parks ($r_{s}=0.261$; sig = 0.000). All correlations are positive, except for the correlations between the variable Residential and all the other variables. Considering the variables belonging to the group intensity of the disease in Poland (see Appendix A, Table A2), there are very significant strong positive correlations between all the pairs of variables new cases, new deaths, and hosp_patients. There are strongly significant weak negative correlations between the variable reproduction rate and the variables new deaths and hosp_patients. There is no correlation between the variable reproduction rate and the variable new cases.

Considering the variables belonging to the group restrictions in Portugal, there is a significant moderate correlation between the two variables stringency_index and new_tests_smoothed ($r_{s}=-0.435$; sig = 0.000). Considering the variables belonging to the group mobility in Portugal (see Appendix A, Table A3), all the correlations are strongly significant. We observe that there are strong to moderate correlations between all the variables, with the exception of a weak correlation between the variables workplaces and parks ($r_{s}=0.260$; sig = 0.000). All correlations are positive, except the correlations between the variable Residential and all the other variables. Considering the variables belonging to the group intensity of the disease in Portugal (see Appendix A Table A4), there are very significant strong positive correlations between all the pairs of variables new cases, new deaths, and hosp_patients. There is a strongly significant weak positive correlation between variable reproduction rate and variable new deaths and a strongly significant weak negative correlation between the variable reproduction rate and the variables hosp_patients. There is no correlation between the variable reproduction rate and the variable new deaths.

### 4.2. Correlations between Variables Indicating Mobility and Restrictions

The correlations between the variables representing restrictions and the variables representing mobility for Poland are presented in Table 3. We can observe that there is a highly significant strong negative correlation between the variable stringency_index and the variables Driving ($r_{s}=-0.710$; sig = 0.000), walking ($r_{s}=-0.793$; sig = 0.000), retail_and_recreation ($r_{s}=-0.648$; sig = 0.000), grocery_and_pharmacy ($r_{s}=-0.527$; sig = 0.000), and transit_stations ($r_{s}=-0.768$; sig = 0.000). There is a strongly significant moderate correlation between the variable stringency_index and the variables parks ($r_{s}=-0.489$; sig = 0.000) and workplaces ($r_{s}=-0.477$; sig = 0.000). There is also a strongly significant strong positive correlation between the variables stringency_index and residential ($r_{s}=0.648$; sig = 0.000).

There are strongly significant weak negative correlations between the variable new_tests_smoothed and the variables driving ($r_{s}=-0.140$; sig = 0.000) and parks ($r_{s}=-0.270$; sig = 0.000). There are strongly significant weak positive correlations between the variable new_tests_smoothed and the variables grocery_and_pharmacy ($r_{s}=0.248$; sig = 0.000), workplaces ($r_{s}=0.250$; sig = 0.000), and Residential ($r_{s}=-0.126$; sig = 0.000). We can consider that there are no correlations between the variable new_tests_smoothed and the variables walking ($r_{s}=0.034$; sig = 0.346), retail_and_recreation ($r_{s}=-0.075$; sig = 0.039), and transit_stations ($r_{s}=0.052$; sig = 0.154).

The correlations between the variables representing restrictions and the variables representing mobility in Portugal are presented in Table 4. First, we can observe that all the correlations for Portugal are strongly significant. There is a strong negative correlation between the variable stringency_index and the variables driving ($r_{s}=-0.692$; sig = 0.000), walking ($r_{s}=-0.805$; sig = 0.000), retail_and_recreation ($r_{s}=-0.742$; sig = 0.000), grocery_and_pharmacy ($r_{s}=-0.671$; sig = 0.000), transit_stations ($r_{s}=-0.833$; sig = 0.000), and workplaces ($r_{s}=-0.582$; sig = 0.000). There is a moderate correlation between the variable stringency_index and the variable parks ($r_{s}=-0.482$; sig = 0.000). There is also a strong negative correlation between the variables stringency_index and residential ($r_{s}=-0.630$; sig = 0.000).

There are strong positive correlations between the variable new_tests_smoothed and the variables walking ($r_{s}=0.525$; sig = 0.000), retail_and_recreation ($r_{s}=0.557$; sig = 0.000), grocery_and_pharmacy ($r_{s}=0.696$; sig = 0.000), and transit_stations ($r_{s}=0.150$; sig = 0.000). There are weak positive correlations between the variable new_tests_smoothed and the variables driving ($r_{s}=0.369$; sig = 0.000), parks ($r_{s}=-0.270$; sig = 0.000), and Workplaces ($r_{s}=0.372$; sig = 0.000). There are weak positive correlations between the variable new_tests_smoothed and the variable residential ($r_{s}=-0.382$; sig = 0.000).

### 4.3. Causations between Variables Indicating Mobility and Restrictions

For calculating causations, we used a Granger causality test. All our variables had a plot time series that showed that neither series was stationary. As a result, we instead studied the first differences of each variable. As we had multiple items for each causation between the group of variables, we tested causation between each pair of items. For group variables of mobility and the restrictions, we had 32 pairs. Each pair represented one hypothesis. Each hypothesis was tested with lags of 1 to 5, 10, 15, 20, 25, and 30 days. We were dealing with multiple testing problems so we used the Bonferroni correction to adjust the *p*-value. The Bonferroni correction sets the significance cut-off at α/n. In the first causations, we had 32 pairs so the corrected *p*-value for significance was a *p*-value < 0.00156.

Causations between the variables representing mobility and the restrictions are presented in Appendix B (Table A5, Table A6, Table A7 and Table A8). We can observe that for Poland, stringency_index does not cause any changes in mobility variables (see Table A5). New_tests_smoothed does not cause any mobility variables (see Table A6). For Portugal, stringency_index does cause retail_and_recreation, grocery_and_pharmacy, and transit for all time lags and driving up to twenty days of lag. Other mobility variables are caused by stringency_index in different time lags (see Table A7). All mobility variables except parks and residential are caused by New_tests_smoothed (see Table A8).

### 4.4. Correlations between Variables Indicating Intensity of the Disease and Restrictions

The correlations between the variables representing restrictions and the variables representing intensity of the disease for Poland are presented in Table 5. First, we can observe that all the correlations for Poland are strongly significant. There are moderate positive correlations between the variable stringency_index and the variables new deaths ($r_{s}=0.354$; sig = 0.000) and hosp_patients ($r_{s}=0.484$; sig = 0.000). There is a weak positive correlation between the variable stringency_index and the variable new cases ($r_{s}=0.166$; sig = 0.000). There is a weak negative correlation between the variable stringency_index and the variable reproduction_rate ($r_{s}=-0.150$; sig = 0.000).

There are strong positive correlations between the variable new_tests_smoothed and the variables new cases ($r_{s}=0.776$; sig = 0.000), new deaths ($r_{s}=0.632$; sig = 0.000), and hosp_patients ($r_{s}=0.724$; sig = 0.000). There is a weak negative correlation between the variable new_tests_smoothed and the variable reproduction rate ($r_{s}=0.166$; sig = 0.000).

The correlations between the variables representing restrictions and the variables representing intensity of the disease for Portugal are presented in Table 6. First, we can observe that all the correlations for Portugal are strongly significant, with the exception of the correlation between the variables new_tests_smoothed and reproduction rate ($r_{s}=-0.004$; sig = 0.901), which have no statistical significance. There are moderate positive correlations between the variable stringency_index and the variables new cases ($r_{s}=0.156$; sig = 0.000), new deaths ($r_{s}=0.193$; sig = 0.000), and hosp_patients ($r_{s}=0.178$; sig = 0.000). There is a weak negative correlation between the variable stringency_index and the variable reproduction rate ($r_{s}=-0.106$; sig = 0.000).

There is a strong positive correlation between the variable new_tests_smoothed and the variable new cases ($r_{s}=0.647$; sig = 0.000). There are weak positive correlations between the variable new_tests_smoothed and the variables new deaths ($r_{s}=0.166$; sig = 0.000) and hosp_patients ($r_{s}=0.305$; sig = 0.000).

### 4.5. Causations between Variables Indicating Intensity of the Disease and Restrictions

For the group variables of the intensity of the disease and the restrictions, we had 16 pairs so the corrected *p*-value for significance was *p*-value < 0.00312. Causations between the variables representing the intensity of the restrictions and restrictions are presented in Appendix B (Table A9, Table A10, Table A11 and Table A12). We can observe for Poland that only hosp_patients caused stringency_index only for 2- and 3-days of lag, while other variables did not cause it (see Table A9). The reproduction rate caused new_tests_smoothed after three days of lag, and new cases and new deaths caused it after ten days of lag (see Table A10). For Portugal, hosp_patients caused stringency_index after two days of lag. New deaths caused it after three days of lag, whereas new cases and the reproduction rate did not affect the stringency index (see Table A11). New deaths and hosp_patients did not have any effect on new_tests_smoothed. New cases caused it after four days of lag and the reproduction rate after ten days (see Table A12).

### 4.6. Correlations between Variables Indicating Mobility and Intensity of the Disease

The correlations between the variables representing mobility and the variables representing intensity of the disease in Poland are presented in Table 7.

There are highly significant strong negative correlations between all the variables indicating mobility and variable hosp_patients ($r_{s}>0.5$; sig = 0.000), except for the variables grocery_and_pharmacy, workplaces, and residential. There is a strongly significant weak negative correlation between the variable grocery_and_pharmacy and the variable hosp_patients ($r_{s}=-0.275$; sig = 0.000), and between the variable workplaces and the variable hosp_patients ($r_{s}=-0.109$; sig = 0.000). There is a very significant strong positive correlation between the variable grocery_and_pharmacy and the variable hosp_patients ($r_{s}=0.644$; sig = 0.000).

There are no correlations or correlations are very weak between all the variables indicating mobility and the variable reproduction rate. As for the variables new cases and new deaths, similar patterns were found. There are strongly significant moderate negative correlations between these two variables and the variables driving, retail_and_recreation, and parks. There is a strongly significant moderate positive correlation between these two variables and the variable Residential. There is no correlation between these two variables and the variable Workplaces.

The correlations between the variables representing mobility and the variables representing intensity of the disease for Portugal are presented in Table 8. There are no correlations between all the variables indicating mobility and the variable reproduction_rate. In addition, there are no correlations between the variable new cases and the variables driving and residential.

There are strongly significant strong negative correlations between the variable parks $(r_{s}=-0.549$; sig = 0.000) and the variables new deaths and hosp_patients $(r_{s}=-0.514$; sig = 0.000). There are strongly significant moderate negative correlations between the variables driving and new deaths $(r_{s}=-0.438$; sig = 0.000), driving and hosp_patients $(r_{s}=-0.392$; sig = 0.000), and walking and new deaths $(r_{s}=-0.336$; sig = 0.000). There are strongly significant moderate positive correlations between the variables grocery_and_pharmacy and new case $(r_{s}=0.330$; sig = 0.000), residential and new deaths $(r_{s}=0.420$; sig = 0.000), and residential and hosp_patients $(r_{s}=0.418$; sig = 0.000). All the other pairs of variables have strongly significant weak positive correlations with variable new cases $(r_{s}\;<\;0.3\;\mathrm{and}>0.1$; sig = 0.000) and strongly significant weak negative correlations with variable new deaths $(r_{s}\;<-0.1\;\mathrm{and}>-0.3$; sig = 0.000).

### 4.7. Causations between Variables Indicating Mobility and Intensity of the Disease

For the group variables of the intensity of the disease and mobility, we had 64 pairs so the corrected *p*-value for significance was p-value < 0.00078. Causations between the variables representing the intensity of the disease and mobility are presented in Appendix B. We can observe for Poland that all of the mobility variables except retail_and_recreation, parks, and transit were caused by new cases in a different time lag but not all the time (see Table A13). We have a similar observation for the variable new_deaths. This variable caused all of the mobility variables except parks (see Table A14). The reproduction rate caused only residential after ten and fifteen days of lag. All other mobility variables were not caused by reproduction rate (see Table A15). Hosp_patients caused, after three days of lag, all mobility variables except parks. Hosp_patients did not cause parks only (see Table A16).

We can observe for Portugal that new cases caused mobility variables such as driving, walking, retail_and_recreation, and grocery_and_pharmacy for time lags but not all the time (except walking). New cases did not cause parks, transit, workplaces, and residential (see Table A17). New deaths and reproduction rate did not cause any changes in the mobility variables (see Table A18 and Table A19). Hosp_patients caused retail_and_recreation and grocery_and_pharmacy but not for all-time lags. For the one-time lag, it caused walking, parks, and residential. Hosp_patients did not cause driving and transit and workplaces (see Table A20).

## 5. Discussion

Our study considers the possibility of a statistical relationship between the emerging dynamics of COVID-19 transmission and mobility in the two countries. Therefore, we took into consideration several publications that address this issue from a data-driven perspective in different countries. We used Google Scholar to search for peer-reviewed articles published in journals relating mobility to coronavirus. The several studies identified from this search concluded that mobility reduction had a positive effect on reducing the transmission of COVID-19. Different data sources were already used to track human mobility during the COVID-19 pandemic, e.g., data points collected from a preinstalled passive Wi-Fi tracking infrastructure [39]; an online questionnaire deployed in many countries asking about transportation [40]; search engine data such as the Baidu Migration Map [41], Google Mobility Reports [42], and Apple Mobility Data [43]; transportation (car, flights) [44,45]; mobile networks operators [46]; the Facebook App [47]; and wearable devices [48]. However, the studies that used data to reach this conclusion were performed using information from the first part of the pandemic, reported in the period from January to July 2020 at the latest. Thus, empirical evidence about the efficacy of social distancing, mobility restriction, and its impact on different aspects of everyday life is still needed to understand its effect on COVID-19 transmission in the two EU countries.

Decreased mobility can be considered as having a significant, positive relationship with the reduced growth of COVID-19 cases. Although social distancing has consistently been shown to have positive effects on COVID-19 transmission in different countries, our work extends these results to the second and third waves of coronavirus. We used real-world mobility data and reported case counts to estimate the relationship between the variables empirically. We also noticed that some studies reported different effects caused by reduced mobility. The reported effects are on economics, social, mental, physical, and governmental aspects. Our results show that social distancing can help to reduce the spread of COVID-19 and should remain a part of personal and institutional responses to the pandemic until countries achieve herd immunity.

Answering RQ1, we noticed a strong correlation between the imposed restrictions and the decreased human mobility reported in Table 3 and Table 4 for both studied countries. Answering RQ2, we noticed that the increase in the level of restrictions imposed by the Government in Poland has a slight positive correlation with the number of new cases, the number of deaths, and the number of hospitalizations, while a slight negative correlation in the reproduction rate of the disease. In contrast, a positive moderate correlation in the restrictions imposed by the State of Portugal is with the number of cases, the number of deaths, and the number of hospitalized patients, while showing a slight negative correlation with the reproduction rate of the disease.

Answering RQ3, we noticed that increased restrictions imposed by the State in Poland and in Portugal have a strong negative correlation with mobility, driving, and walking, mainly in places related to retail and recreation, grocery and pharmacy, and transit stations but also a moderate negative correlation in mobility related to parks and workplaces. However, the causation does not confirm this result for Poland, only for Portugal. We can conclude that restriction measures imposed by the State of Poland and of Portugal are highly negatively correlated with the mobility of citizens, except those related to the residential which increased. Answering RQ4, we noticed that the increase in restrictions is highly positively correlated to stays in residential places both in Poland and Portugal.

Empirical studies are needed to gain knowledge about the basis of this phenomenon and the observable facts resulting from the analysis of data on human movement during the COVID-19 pandemic and after the introduction of nonpharmaceutical interventions. It is also necessary to thoroughly analyze the literature, which presents many approaches to the researched topic, including various models and complex databases [49]. Research on the impact of mobility on the development of the COVID-19 pandemic, as well as the analysis of travel data and presentation of the conclusions, may have an indirect impact on the decisions of the inhabitants of particular regions. There is a probability that the conclusions confirmed by reliable research may have an impact on the deliberate limitation of mobility as well as demonstrate the impact of nonpharmaceutical interventions on the pandemic development. In the course of the research, data on mobility were analyzed in the context of the attitude of citizens and their obedience to regulations, despite whether the changes in mobility resulted from governmental restrictions or were undertaken voluntarily as a result of social solidarity. Proving whether the restriction of mobility was followed is an important factor to understand the impact of the pandemic on the economic decision-making of individuals.

### Study Limitations

Research centers and enterprises have joined the fight against the global pandemic not only by conducting research but also by monitoring mobility, the restrictions introduced [50], and their impact on the spread of the virus. However, it cannot be denied that accurate mobility data are hard to obtain [8], and they are often limited and incomplete. This is because the data on mobility only concerns people who use mobile phones daily [51], with their location enabled. In some countries, owning a mobile phone or a smartphone is not yet popular. In addition, people, e.g., the homeless or those in a bad economic situation, do not have a phone. Furthermore, the elderly often use simplified telephones that do not use the location service. Google and Apple share reports on the mobility of the population, using new technologies that are an integral part of their products or services daily. Despite the aforementioned limitations, these data cover a large proportion of the population and can be considered to provide results.

From the literature research, we derived risk analysis and several known limitations in our research. First, we are aware that our sample cannot be considered as a representative sample of the whole population [52]. Second, countries may offer fallible reporting on COVID-19 mortality rates. Many unmeasured NPI, other than human mobility, can reduce mortality during the pandemic, including case surveillance, contact tracing, adequate personal protective equipment, and the regularity of hand hygiene [53,54]. Third, many changes in the observed mobility resulted from government restrictions [55], and there were behavioral limitations imposed by the policies of several European countries where the COVID-19 infection had rapidly spread [56].

Fourth, the collected data might have errors due to both reporting issues and limited testing capacity [46] and certainly does not represent the real number of people going outside [57]. Children and the elderly are frequently under-represented in mobile phone data, and inferences derived from mobile phone users may not be generalized to these populations [58]. An analysis by age group or gender to explore the sensitive population could not be conducted due to the lack of related information [57]. Fifth, Apple and Google mobility data are based on product usage within the population. There is variability in volume, consistent with normal, seasonal usage, the large dataset, and the diversity of climates. Further, the penetration of these products into a particular market and its usage bias towards certain population groups (e.g., younger people, greater economic capital) may have influenced the results [43].

Seventh, although causal-comparative research effectively establishes relationships between variables, there are many limitations to this type of research. As causal-comparative research occurs ex post facto, we have no control over the variables and thus cannot manipulate them. In addition, there are often variables other than what we selected for this study that may impact the mobility restrictions. Reversal causation is another issue that may arise in causal-comparative research.

## 6. Conclusions

The results of the study consist in revealing (a) the impact of the NPI on changes in human mobility during the coronavirus pandemic, and (b) the correlation and causation between these changes and the spread of the virus. We assume that the reaction of people to the NPI in European Union countries slightly differed; in some countries, people stayed at home in 100% of cases, while in other countries, there were people who rejected the necessity to limit their movements. The difference might be explained not only by individual reactions but also by the severity of the restrictions in a particular country. We see the contribution of the research to the global struggle with the COVID-19 pandemic in two major issues. First, we believe that the results of the analysis of the impact of human mobility on coronavirus infection spread will allow people to become more aware of the necessity of obeying the restrictions, keeping social distanced, and staying at home. Second, we consider the model of correlation and causation between human mobility and the COVID-19 infection rate to be useful as an additional tool for analyzing and preventing the virus spread. In addition to that, the application of the selected set of methods of data analysis and processing to the specific set of statistical data is considered a contribution to science in general.

## Figures and Tables

**Table 1:** Dataset with sources and indicator descriptions.

Group of Indicators	Source	Indicator	Description
Vaccination Rates	HDX [36]	total_vaccinations_per_hundred	Total number of COVID-19 vaccination doses administered per 100 people in the total population
		people_vaccinated_per_hundred	Total number of people who received at least one vaccine dose per 100 people in the total population
		people_fully_vaccinated_per_hundred	Total number of people who received all doses prescribed by the initial vaccination protocol per 100 people in the total population
		total_boosters_per_hundred	Total number of COVID-19 vaccination booster doses administered per 100 people in the total population
		new_vaccinations_smoothed_per_million	New COVID-19 vaccination doses administered (7-day smoothed) per 1,000,000 people in the total population
Restrictions	HDX [36]	stringency_index	Government Response Stringency Index: composite measure based on 9 response indicators including school closures, workplace closures, and travel bans, rescaled to a value from 0 to 100 (100 = strictest response)
		new_tests_smoothed	New tests for COVID-19 (7-day smoothed)
Mobility	Apple Mobility Report [35]	driving	Mobility trends of people making requests to Apple Maps for driving directions
		walking	Mobility trends of people making requests to Apple Maps for walking directions
	Google Mobility Data [34]	retail_and_recreation	Mobility trends for places such as restaurants, cafes, shopping centers, theme parks, museums, libraries, and movie theaters
		grocery_and_pharmacy	Mobility trends for places such as grocery markets, food warehouses, farmers markets, specialty food shops, drug stores, and pharmacies
		parks	Mobility trends for places such as national parks, public beaches, marinas, dog parks, plazas, and public gardens
		transit_stations	Mobility trends for places such as public transport hubs, e.g., subway, bus, and train stations
		workplaces	Mobility trends for places of work
		residential	Mobility trends for places of residence
Intensity	HDX [36]	new cases	New confirmed cases of COVID-19
		new deaths	New deaths attributed to COVID-19
		reproduction rate	Real-time estimate of the effective reproduction rate (R) of COVID-19
		hosp_patients	Number of COVID-19 patients in hospital on a given day

**Table 2:** Resume of the means of the vaccination variables concerning Portugal and Poland.

Variables	Location	N	Mean	Std. Deviation	Std. Error Mean
total_vaccinations_per_hundred	Poland	401	80.2226	49.14909	2.45439
	Portugal	366	99.4016	79.50633	4.15586
people_vaccinated_per_hundred	Poland	399	39.9841	21.11410	1.05703
	Portugal	279	41.0038	33.35521	1.99692
people_fully_vaccinated_per_hundred	Poland	382	37.7337	21.98770	1.12499
	Portugal	340	46.0523	37.41164	2.02893
total_boosters_per_hundred	Poland	153	20.8429	10.28417	0.83143
	Portugal	157	25.2122	23.71572	1.89272
new_vaccinations_smoothed_per_million	Poland	483	2968.37	2354.947	107.154
	Portugal	438	5146.66	3363.988	160.738

**Table 3:** Spearman’s *r_s_* correlation indexes calculated between variables indicating restrictions and mobility in Poland.

Variables	stringency_index	new_tests_smoothed
driving	−0.710 **	−0.140 **
walking	−0.793 **	0.034
retail_and_recreation	−0.648 **	−0.075 *
grocery_and_pharmacy	−0.527 **	0.248 **
parks	−0.489 **	−0.270 **
transit_stations	−0.768 **	0.052
workplaces	−0.477 **	0.250 **
residential	0.648 **	0.126 **

** Correlation is significant at the 0.01 level (two-tailed). * Correlation is significant at the 0.05 level (two-tailed).

**Table 4:** Spearman’s *r_s_* correlation indexes calculated between variables indicating restrictions and mobility in Portugal.

Variables	stringency_index	new_tests_smoothed
driving	−0.692 **	0.369 **
walking	−0.805 **	0.525 **
retail_and_recreation	−0.742 **	0.557 **
grocery_and_pharmacy	−0.671 **	0.696 **
parks	−0.482 **	0.150 **
transit_stations	−0.833 **	0.563 **
workplaces	−0.586 **	0.372 **
residential	0.630 **	−0.382 **

** Correlation is significant at the 0.01 level (two-tailed).

**Table 5:** Spearman’s *r_s_* correlation indexes calculated between variables indicating restrictions and intensity of the disease for Poland.

Variables	stringency_index	new_tests_smoothed
new cases	0.166 **	0.776 **
new deaths	0.354 **	0.632 **
reproduction rate	−0.150 **	−0.193 **
hosp_patients	0.484 **	0.724 **

** Correlation is significant at the 0.01 level (two-tailed).

**Table 6:** Spearman’s *r_s_* correlation indexes calculated between variables indicating restrictions and intensity of the disease in Poland.

Variables	stringency_index	new_tests_smoothed
new cases	0.156 **	0.647 **
new deaths	0.193 **	0.166 **
reproduction rate	−0.106 **	−0.004
hosp_patients	0.178 **	0.305 **

** Correlation is significant at the 0.01 level (two-tailed).

**Table 7:** Spearman’s *r_s_* correlation indexes calculated between variables indicating mobility and intensity of the disease in Poland.

Variables	New Cases	New Deaths	Reproduction Rate	hosp_patients
driving	−0.350 **	−0.430 **	−0.069	−0.699 **
walking	−0.177 **	−0.298 **	0.008	−0.563 **
retail_and_recreation	−0.361 **	−0.386 **	−0.083 *	−0.630 **
grocery_and_pharmacy	−0.039	−0.133 **	−0.113 **	−0.275 **
parks	−0.496 **	−0.491 **	−0.120 **	−0.731 **
transit_stations	−0.232 **	−0.325 **	−0.045	−0.537 **
workplaces	0.077 *	−0.045	−0.104 **	−0.109 **
residential	0.370 **	0.423 **	0.030	0.644 **

** Correlation is significant at the 0.01 level (two-tailed). * Correlation is significant at the 0.05 level (two-tailed).

**Table 8:** Spearman’s *r_s_* correlation indexes calculated between variables indicating mobility and intensity of the disease for Portugal.

Variables	New Cases	New Deaths	Reproduction Rate	hosp_patients
driving	0.004	−0.438 **	−0.047	−0.392 **
walking	0.129 **	−0.336 **	−0.030	−0.292 **
retail_and_recreation	0.189 **	−0.272 **	0.036	−0.258 **
grocery_and_pharmacy	0.330 **	−0.168 **	0.018	−0.125 **
parks	−0.184 **	−0.549 **	−0.025	−0.514 **
transit_stations	0.168 **	−0.286 **	0.044	−0.268 **
workplaces	0.106 **	−0.218 **	0.078 *	−0.183 **
residential	−0.006	0.420 **	−0.056	0.418 **

** Correlation is significant at the 0.01 level (two-tailed). * Correlation is significant at the 0.05 level (two-tailed).

## Data Availability

The data presented in this study are available in Supplementary Material.

## Supplementary Materials

The following supporting information can be downloaded at: https://www.mdpi.com/article/10.3390/ijerph192114455/s1.

## Appendix A

**Table A1:** Correlations between variables belonging to group Mobility in Poland.

Variables	Driving	Walking	Retail_and_Recreation	Grocery_and_Pharmacy	Parks	Transit_Stations	Workplaces	Residential
driving	-	0.914 **	0.862 **	0.644 **	0.833 **	0.813 **	0.367 **	−0.803 **
walking	0.914 **	-	0.808 **	0.690 **	0.723 **	0.855 **	0.447 **	−0.758 **
retail_and_recreation	0.862 **	0.808 **	-	0.792 **	0.734 **	0.857 **	0.413 **	−0.773 **
grocery_and_pharmacy	0.644 **	0.690 **	0.792 **	-	0.481 **	0.790 **	0.525 **	−0.603 **
parks	0.833 **	0.723 **	0.734 **	0.481 **	-	0.680 **	0.261 **	−0.768 **
transit_stations	0.813 **	0.855 **	0.857 **	0.790 **	0.680 **	-	0.721 **	−0.889 **
workplaces	0.367 **	0.447 **	0.413 **	0.525 **	0.261 **	0.721 **	-	−0.681 **
residential	−0.803 **	−0.758 **	−0.773 **	−0.603 **	−0.768 **	−0.889 **	−0.681 **	-

** Correlation is significant at the 0.01 level (two-tailed).

**Table A2:** Correlations between variables belonging to group Mobility in Portugal.

Variables	Driving	Walking	Retail_and_Recreation	Grocery_and_Pharmacy	Parks	Transit_Stations	Workplaces	Residential
driving	-	0.924 **	0.839 **	0.705 **	0.858 **	0.825 **	0.487 **	−0.759 **
walking	0.924 **	-	0.897 **	0.806 **	0.717 **	0.910 **	0.548 **	−0.754 **
retail_and_recreation	0.839 **	0.897 **	-	0.919 **	0.722 **	0.886 **	0.440 **	−0.698 **
grocery_and_pharmacy	0.705 **	0.806 **	0.919 **	-	0.555 **	0.816 **	0.386 **	−0.579 **
parks	0.858 **	0.717 **	0.722 **	0.555 **	-	0.603 **	0.260 **	−0.607 **
transit_stations	0.825 **	0.910 **	0.886 **	0.816 **	0.603 **	-	0.732 **	−0.865 **
workplaces	0.487 **	0.548 **	0.440 **	0.386 **	0.260 **	0.732 **	-	−0.852 **
residential	−0.759 **	−0.754 **	−0.698 **	−0.579 **	−0.607 **	−0.865 **	−0.852 **	-

** Correlation is significant at the 0.01 level (two-tailed).

**Table A3:** Correlations between variables belonging to group Intensity of the disease in Poland.

Variables	New Cases	New Deaths	Reproduction Rate	hosp_patients
new cases	-	0.807 **	0.034	0.870 **
new deaths	0.807 **	-	−0.215 **	0.825 **
reproduction rate	0.034	−0.215 **	-	−0.198 **
hosp_patients	0.870 **	0.825 **	−0.198 **	-

** Correlation is significant at the 0.01 level (two-tailed).

**Table A4:** Correlations between variables belonging to group Intensity of the disease in Portugal.

Variables	New Cases	New Deaths	Reproduction Rate	hosp_patients
new cases	-	0.704 **	0.178 **	0.708 **
new deaths	0.704 **	-	−0.087 *	0.892 **
reproduction rate	0.178 **	−0.087 *	-	−0.165 **
hosp_patients	0.708 **	0.892 **	−0.165 **	-

** Correlation is significant at the 0.01 level (two-tailed). * Correlation is significant at the 0.05 level (two-tailed).

## Appendix B

**Table A5:** Stringency_index (Str), Granger-cause mobility variables, and *p*-values in day lags in Poland.

Day Lag	Str->Driving	Str->Walking	Str->Retail	Str->Grocery	Str->Parks	Str->Transit	Str->Workplaces	Str->Residential
1	0.924	0.434	0.099	0.139	0.864	0.936	0.538	0.883
2	0.917	0.733	0.113	0.298	0.240	0.555	0.772	0.653
3	0.496	0.542	0.154	0.451	0.307	0.695	0.822	0.822
4	0.407	0.611	0.062	0.437	0.504	0.701	0.863	0.840
5	0.586	0.536	0.112	0.572	0.535	0.273	0.453	0.274
10	0.281	0.017	0.253	0.965	0.743	0.295	0.879	0.627
15	0.087	0.056	0.536	0.997	0.681	0.437	0.839	0.625
20	0.223	0.055	0.316	0.948	0.646	0.027	0.061	0.021
25	0.291	0.170	0.641	0.993	0.894	0.225	0.171	0.075
30	0.438	0.520	0.830	0.997	0.981	0.402	0.212	0.125

Significant at Bonferroni corrected *p* < 0.00156.

**Table A6:** New_tests_smoothed (NTS), Granger-cause mobility variables, and *p*-values in day lags in Poland.

Day Lag	NTS->Driving	NTS->Walking	NTS->Retail	NTS->Grocery	NTS->Parks	NTS->Transit	NTS->Workplaces	NTS->Residential
1	0.911	0.994	0.955	0.919	0.813	0.845	0.895	0.872
2	0.761	0.842	0.645	0.775	0.841	0.916	0.758	0.843
3	0.622	0.781	0.865	0.975	0.961	0.957	0.905	0.870
4	0.758	0.883	0.942	0.992	0.944	0.944	0.904	0.912
5	0.462	0.594	0.889	0.995	0.966	0.958	0.850	0.709
10	0.622	0.341	0.652	0.841	0.998	0.860	0.098	0.100
15	0.817	0.637	0.700	0.846	1.000	0.822	0.231	0.194
20	0.976	0.843	0.864	0.590	1.000	0.873	0.272	0.201
25	0.998	0.972	0.991	0.689	1.000	0.946	0.420	0.209
30	0.999	0.989	0.961	0.676	1.000	0.878	0.554	0.416

Significant at Bonferroni corrected *p* < 0.00156.

**Table A7:** Stringency_index (Str), Granger-cause mobility variables, and *p*-values in day lags in Portugal.

Day Lag	Str->Driving	Str->Walking	Str->Retail	Str->Grocery	Str->Parks	Str->Transit	Str->Workplaces	Str->Residential
1	0.000 *	0.625	0.000 *	0.000 *	0.001 *	0.000 *	0.188	0.585
2	0.000 *	0.043	0.000 *	0.000 *	0.001 *	0.000 *	0.316	0.341
3	0.000 *	0.131	0.000 *	0.000 *	0.005 *	0.000 *	0.498	0.606
4	0.000 *	0.075	0.000 *	0.000 *	0.015	0.000 *	0.749	0.758
5	0.001 *	0.211	0.001 *	0.000 *	0.045	0.000 *	0.292	0.202
10	0.000 *	0.504	0.000 *	0.000 *	0.040	0.000 *	0.083	0.000 *
15	0.001 *	0.620	0.000 *	0.000 *	0.173	0.000 *	0.030	0.000 *
20	0.001 *	0.720	0.000 *	0.000 *	0.256	0.000 *	0.031	0.000 *
25	0.017	0.911	0.000 *	0.000 *	0.367	0.000 *	0.027	0.000 *
30	0.033	0.903	0.000 *	0.000 *	0.521	0.001 *	0.063	0.000 *

* Significant at Bonferroni corrected *p* < 0.00156.

**Table A8:** New_tests_smoothed (NTS), Granger-cause mobility variables, and *p*-values in day lags in Portugal.

Day Lag	NTS->Driving	NTS->Walking	NTS->Retail	NTS->Grocery	NTS->Parks	NTS->Transit	NTS->Workplaces	NTS->Residential
1	0.000 *	0.001 *	0.000 *	0.015	0.295	0.000 *	0.001 *	0.024
2	0.001 *	0.001 *	0.006	0.022	0.483	0.000 *	0.010	0.100
3	0.002	0.004	0.017	0.155	0.734	0.003	0.034	0.185
4	0.002	0.005	0.007	0.240	0.627	0.002	0.055	0.279
5	0.001 *	0.000 *	0.003	0.097	0.730	0.000 *	0.021	0.186
10	0.000 *	0.000 *	0.000 *	0.000 *	0.532	0.000 *	0.000 *	0.258
15	0.000 *	0.000 *	0.000 *	0.000 *	0.138	0.000 *	0.000 *	0.061
20	0.000 *	0.000 *	0.000 *	0.000 *	0.139	0.000 *	0.003 *	0.172
25	0.000 *	0.000 *	0.000 *	0.000 *	0.208	0.000 *	0.017	0.366
30	0.000 *	0.000 *	0.000 *	0.000 *	0.304	0.003	0.041	0.747

* Significant at Bonferroni corrected *p* < 0.00156.

**Table A9:** Intensity variables, Granger-cause Stringency_index (Str), and *p*-values in day lags in Poland.

Day Lag	New Cases->Str	New Deaths->Str	Reproduction Rate->Str	Hosp Patients->Str
1	0.124	0.412	0.780	0.016
2	0.135	0.598	0.429	0.001 *
3	0.123	0.670	0.230	0.005 *
4	0.139	0.501	0.262	0.003
5	0.201	0.614	0.202	0.009
10	0.339	0.361	0.401	0.008
15	0.744	0.597	0.111	0.098
20	0.848	0.700	0.165	0.117
25	0.931	0.883	0.373	0.211
30	0.895	0.950	0.301	0.332

* Significant at Bonferroni corrected *p* < 0.00312.

**Table A10:** Intensity variables, Granger-cause New_tests_smoothed (NTS), and *p*-values in day lags in Poland.

Day Lag	New Cases->NTS	New Deaths->NTS	Reproduction Rate->NTS	Hosp Patients->NTS
1	0.170	0.096	0.043	0.625
2	0.172	0.006	0.012	0.540
3	0.391	0.021	0.002 *	0.754
4	0.067	0.004	0.001 *	0.154
5	0.005	0.009	0.002 *	0.241
10	0.000 *	0.000 *	0.000 *	0.123
15	0.000 *	0.001 *	0.000 *	0.253
20	0.001 *	0.000 *	0.001 *	0.168
25	0.003 *	0.000 *	0.003	0.091
30	0.019	0.001 *	0.008	0.196

* Significant at Bonferroni corrected *p* < 0.00312.

**Table A11:** Intensity variables, Granger-cause Stringency_index (Str), and *p*-values in day lags in Portugal.

Day Lag	New Cases->Str	New Deaths->Str	Reproduction Rate->Str	Hosp Patients->Str
1	0.238	0.138	0.406	0.004
2	0.151	0.288	0.542	0.000 *
3	0.052	0.000 *	0.019	0.000 *
4	0.167	0.001 *	0.014	0.000 *
5	0.372	0.001 *	0.029	0.000 *
10	0.902	0.000 *	0.143	0.000 *
15	0.781	0.000 *	0.098	0.000 *
20	0.644	0.000 *	0.025	0.000 *
25	0.666	0.000 *	0.054	0.000 *
30	0.630	0.001 *	0.088	0.000 *

* Significant at Bonferroni corrected *p* < 0.00312.

**Table A12:** Intensity variables, Granger-cause New_tests_smoothed (NTS), and *p*-values in day lags in Portugal.

Day Lag	New Cases->NTS	New Deaths->NTS	Reproduction Rate->NTS	Hosp Patients->NTS
1	0.307	0.457	0.049	0.201
2	0.381	0.500	0.034	0.302
3	0.006	0.503	0.040	0.682
4	0.000 *	0.182	0.017	0.848
5	0.000 *	0.263	0.022	0.916
10	0.000 *	0.819	0.002 *	0.804
15	0.000 *	0.892	0.001 *	0.800
20	0.000 *	0.815	0.001 *	0.930
25	0.000 *	0.673	0.001 *	0.956
30	0.000 *	0.826	0.002 *	0.970

* Significant at Bonferroni corrected *p* < 0.00312.

**Table A13:** New_cases (NC), Granger-cause mobility variables, and *p*-values in day lags in Poland.

Day Lag	NC->Driving	NC->Walking	NC->Retail	NC->Grocery	NC->Parks	NC->Transit	NC->Workplaces	NC->Residential
1	0.907	0.896	0.024	0.069	0.567	0.516	0.886	0.810
2	0.089	0.000 *	0.011	0.722	0.832	0.559	0.271	0.795
3	0.002	0.000 *	0.001	0.953	0.887	0.527	0.000 *	0.002
4	0.000 *	0.000 *	0.010	0.000 *	0.779	0.436	0.000 *	0.000 *
5	0.000 *	0.000 *	0.239	0.000 *	0.720	0.318	0.000 *	0.000 *
10	0.000 *	0.000 *	0.380	0.006	0.887	0.015	0.000 *	0.002
15	0.000 *	0.004	0.119	0.009	0.921	0.026	0.007	0.009
20	0.000 *	0.016	0.247	0.014	0.888	0.051	0.070	0.028
25	0.001	0.056	0.348	0.002	0.908	0.030	0.115	0.065
30	0.001	0.070	0.392	0.002	0.962	0.042	0.049	0.035

* Significant at Bonferroni corrected *p* < 0.00078.

**Table A14:** New_deaths (ND), Granger-cause mobility variables, and *p*-values in day lags in Poland.

Day Lag	ND->Driving	ND->Walking	ND->Retail	ND->Grocery	ND->Parks	ND->Transit	ND->Workplaces	ND->Residential
1	0.219	0.103	0.004	0.060	0.016	0.672	0.126	0.139
2	0.471	0.000 *	0.003	0.819	0.472	0.106	0.267	0.572
3	0.001 *	0.000 *	0.002	0.399	0.339	0.052	0.000 *	0.008 *
4	0.000 *	0.000 *	0.001	0.210	0.056	0.012	0.000 *	0.000 *
5	0.000 *	0.000 *	0.056	0.000 *	0.149	0.014	0.000 *	0.000 *
10	0.000 *	0.000 *	0.408	0.001	0.243	0.027	0.000 *	0.000 *
15	0.000 *	0.000 *	0.000 *	0.000 *	0.588	0.000 *	0.000 *	0.000 *
20	0.000 *	0.000 *	0.000 *	0.000 *	0.619	0.000 *	0.000 *	0.000 *
25	0.000 *	0.000 *	0.000 *	0.000 *	0.339	0.000 *	0.000 *	0.000 *
30	0.000 *	0.000 *	0.000 *	0.000 *	0.400	0.000 *	0.000 *	0.000 *

* Significant at Bonferroni corrected *p* < 0.00078.

**Table A15:** Reproduction rate (RR), Granger-cause mobility variables, and *p*-values in day lags in Poland.

Day Lag	RR->Driving	RR->Walking	RR->Retail	RR->Grocery	RR->Parks	RR->Transit	RR->Workplaces	RR->Residential
1	0.262	0.799	0.735	0.374	0.400	0.605	0.512	0.211
2	0.489	0.570	0.012	0.048	0.576	0.091	0.078	0.248
3	0.632	0.666	0.011	0.032	0.742	0.047 *	0.040	0.268
4	0.731	0.733	0.052	0.056	0.886	0.102	0.136	0.497
5	0.908	0.884	0.135	0.083	0.953	0.327	0.076	0.654
10	0.100	0.568	0.419	0.183	0.518	0.070	0.000 *	0.007
15	0.004	0.423	0.216	0.233	0.780	0.010	0.000 *	0.004
20	0.009	0.424	0.344	0.599	0.610	0.034	0.005	0.013
25	0.008	0.508	0.627	0.478	0.616	0.016	0.014	0.016
30	0.014	0.531	0.567	0.419	0.612	0.015	0.037	0.052

* Significant at Bonferroni corrected *p* < 0.00078.

**Table A16:** Hosp_patients (HP), Granger-cause mobility variables, and *p*-values in day lags in Poland.

Day Lag	HP->Driving	HP->Walking	HP->Retail	HP->Grocery	HP->Parks	HP->Transit	HP->Workplaces	HP->Residential
1	0.414	0.498	0.303	0.618	0.891	0.987	0.959	0.751
2	0.089	0.344	0.000 *	0.308	0.865	0.083	0.085	0.395
3	0.000 *	0.000 *	0.007	0.079	0.523	0.103	0.036	0.274
4	0.000 *	0.000 *	0.000 *	0.002	0.244	0.192	0.031	0.033
5	0.000 *	0.000 *	0.000 *	0.000 *	0.188	0.021	0.000 *	0.000 *
10	0.000 *	0.000 *	0.054	0.067	0.478	0.011	0.000 *	0.000 *
15	0.000 *	0.000 *	0.016	0.056	0.578	0.000 *	0.000 *	0.000 *
20	0.000 *	0.000 *	0.009	0.008	0.697	0.000 *	0.000 *	0.000 *
25	0.000 *	0.000 *	0.011	0.000 *	0.606	0.000 *	0.000 *	0.000 *
30	0.000 *	0.008	0.027	0.005	0.600	0.000 *	0.000 *	0.000 *

* Significant at Bonferroni corrected *p* < 0.00078.

**Table A17:** New_cases (NC), Granger-cause mobility variables, and *p*-values in day lags in Portugal.

Day Lag	NC->Driving	NC->Walking	NC->Retail	NC->Grocery	NC->Parks	NC->Transit	NC->Workplaces	NC->Residential
1	0.545	0.388	0.006	0.001 *	0.746	0.211	0.559	0.479
2	0.428	0.000 *	0.006	0.044	0.224	0.105	0.845	0.911
3	0.033	0.000 *	0.000 *	0.000 *	0.045	0.165	0.409	0.751
4	0.001	0.000 *	0.000 *	0.000 *	0.218	0.485	0.006	0.029
5	0.000 *	0.000 *	0.047	0.000 *	0.668	0.711	0.141	0.179
10	0.036	0.000 *	0.480	0.408	0.852	0.635	0.323	0.536
15	0.122	0.000 *	0.591	0.493	0.956	0.846	0.757	0.839
20	0.104	0.000 *	0.700	0.806	0.979	0.968	0.985	0.985
25	0.311	0.000 *	0.853	0.753	0.993	0.974	0.998	0.989
30	0.268	0.000 *	0.341	0.190	0.990	0.790	0.998	0.951

* Significant at Bonferroni corrected *p* < 0.00078.

**Table A18:** New_deaths (ND), Granger-cause mobility variables, and *p*-values in day lags in Portugal.

Day Lag	ND->Driving	ND->Walking	ND->Retail	ND->Grocery	ND->Parks	ND->Transit	ND->Workplaces	ND->Residential
1	0.663	0.428	0.896	0.891	0.622	0.442	0.361	0.167
2	0.191	0.728	0.329	0.165	0.875	0.117	0.766	0.382
3	0.312	0.905	0.146	0.299	0.389	0.152	0.833	0.189
4	0.258	0.692	0.336	0.333	0.204	0.275	0.557	0.197
5	0.191	0.742	0.214	0.191	0.362	0.095	0.824	0.176
10	0.831	0.586	0.412	0.237	0.691	0.423	0.830	0.272
15	0.951	0.643	0.476	0.170	0.603	0.159	0.464	0.064
20	0.960	0.858	0.405	0.060	0.617	0.289	0.398	0.064
25	0.894	0.834	0.407	0.190	0.593	0.652	0.517	0.173
30	0.917	0.863	0.174	0.093	0.840	0.807	0.704	0.340

Significant at Bonferroni corrected *p* < 0.00078.

**Table A19:** Reproduction rate (RR), Granger-cause mobility variables, and *p*-values in day lags in Portugal.

Day Lag	RR->Driving	RR->Walking	RR->Retail	RR->Grocery	RR->Parks	RR->Transit	RR->Workplaces	RR->Residential
1	0.501	0.390	0.885	0.955	0.435	0.702	0.577	0.712
2	0.399	0.745	0.244	0.578	0.290	0.167	0.472	0.196
3	0.727	0.047	0.441	0.467	0.326	0.389	0.212	0.017
4	0.066	0.035	0.108	0.059	0.386	0.481	0.208	0.023
5	0.302	0.173	0.469	0.291	0.385	0.767	0.689	0.263
10	0.154	0.558	0.182	0.062	0.534	0.929	0.602	0.990
15	0.168	0.288	0.189	0.030	0.570	0.640	0.096	0.859
20	0.005	0.011	0.026	0.039	0.317	0.256	0.459	0.800
25	0.004	0.003	0.003	0.002	0.264	0.364	0.733	0.906
30	0.007	0.002	0.004	0.003	0.207	0.071	0.271	0.506

Significant at Bonferroni corrected *p* < 0.00078.

**Table A20:** Hosp_patients (HP), Granger-cause mobility variables, and *p*-values in day lags in Portugal.

Day Lag	HP->Driving	HP->Walking	HP->Retail	HP->Grocery	HP->Parks	HP->Transit	HP->Workplaces	HP->Residential
1	0.187	0.504	0.000 *	0.000 *	0.004	0.134	0.001	0.002
2	0.964	0.690	0.000 *	0.000 *	0.005	0.408	0.008	0.006
3	0.808	0.534	0.000 *	0.000 *	0.014	0.758	0.033	0.016
4	0.036	0.173	0.000 *	0.002	0.004	0.126	0.024	0.001
5	0.131	0.000 *	0.000 *	0.010	0.000 *	0.143	0.022	0.001
10	0.060	0.229	0.001	0.007	0.027	0.192	0.203	0.072
15	0.031	0.362	0.012	0.017	0.553	0.155	0.365	0.113
20	0.066	0.462	0.002	0.000 *	0.654	0.074	0.451	0.084
25	0.039	0.423	0.000 *	0.000 *	0.578	0.017	0.367	0.004
30	0.034	0.538	0.000 *	0.000 *	0.807	0.044	0.336	0.007

* Significant at Bonferroni corrected *p* < 0.00078.
